# Engineering Viral Surface Antigens to Improve Display on Virus-like Particle (VLP) Vaccine Prototypes

**DOI:** 10.3390/biotech15020038

**Published:** 2026-05-27

**Authors:** Mona Pißarreck, Kristina Katsoutas, Jörn Stitz

**Affiliations:** 1 Research Group Pharmaceutical Biotechnology, Faculty of Applied Natural Sciences, TH Köln University of Applied Sciences, Campusplatz 1, 51379 Leverkusen, Germany; mona.pissarreck@th-koeln.de (M.P.); kristina.katsoutas@th-koeln.de (K.K.); 2 Institute of Biology, Faculty IV: School of Science and Technology, University of Siegen, 57068 Siegen, Germany

**Keywords:** virus-like particles, vaccine, SARS-CoV-2 spike protein, HIV Gag, extracellular particles, protein engineering, cytoplasmic tail

## Abstract

**Objectives**: Membrane-enveloped virus-like particles (VLPs) constitute a versatile vaccine platform allowing for the display of heterologous viral surface antigens. The density of displayed antigens is paramount for the efficient elicitation of a strong cellular and humoral immune response. SARS-CoV-2 spike protein variants with engineered cytoplasmic tails (CTs) were generated to enhance decoration efficiency on the surface of VLPs formed by the HIV core protein Gag. These HIV (SARS-CoV-2) chimeric particles serve as a vaccine component prototype. **Methods**: Spike variants were first analyzed for cellular and surface expression as well as incorporation into extracellular vesicles (EVs) and VLPs using flow cytometric analysis and Western blot analysis. Receptor binding, fusogenicity, i.e., mediating the fusion of spike-positive with receptor-containing membranes, and the proteins’ potential to mediate lentiviral vector gene transduction into susceptible target cells was examined by employing syncytia-formation assays and vector titration experiments. The display of a neutralization-sensitive epitope was examined utilizing immuno-precipitation using a neutralizing antibody. **Results**: All four variants were shown to be cell-surface expressed, to recruit the cognate receptor, to mediate membrane fusion and cell entry of lentiviral pseudotype vector particles and to decorate VLPs and EVs. However, the spike variant encompassing a truncated CT derived from the gibbon ape leukemia virus (GaLV) transmembrane (TM) envelope protein was most efficiently incorporated into HIV Gag-formed VLPs. All variants exposed a neutralization-sensitive epitope in the receptor binding domain. **Conclusions**: Engineering of the CTs of viral surface antigens can enhance VLP decoration, while required functionality of the ecto-domain such as receptor recognition, fusogenicity and neutralization-sensitive epitope presentation are not abrogated. This indicates the preservation of the structural integrity of the antigen required to elicit a neutralizing humoral immunity upon vaccination. The identified truncated CT of GaLV TM may be of utility to improve the incorporation of other viral surface antigens into a variety of membrane-enveloped VLPs derived from a range of different parental viruses.

## 1. Introduction

Virus-like particles (VLPs) are virus-derived nanoparticles that retain the morphology and size of the parental virus while lacking a viral genome. VLPs are thus replication-deficient and non-infectious, providing a high bio-safety profile and rendering these particulates a preferred and advanced vaccine platform. VLP vaccines can be divided into two different phenotypes, namely, capsid particles and membrane-enveloped particles. Examples of parental capsid viruses of VLPs include poliovirus ([1], reviewed by Han et al. [2]), causing paralysis in children, and human papilloma virus (HPV), inducing a variety of tumors (reviewed by Tommasino [3] and Zhang et al. [4]). The HPV VLP vaccines Gardasil™ and Cevarix™ are already approved and part of an adolescent vaccination program in many countries [5,6,7]. In contrast, VLPs can be enveloped by the membrane taken from the producing cell during egress or budding of the newly formed particles. Amongst others, enveloped VLPs were derived from influenza virus, SARS-CoV-2, respiratory syncytial virus (RSV) and HIV-1, to name a few [8,9,10,11,12,13].

**Figure 1:**
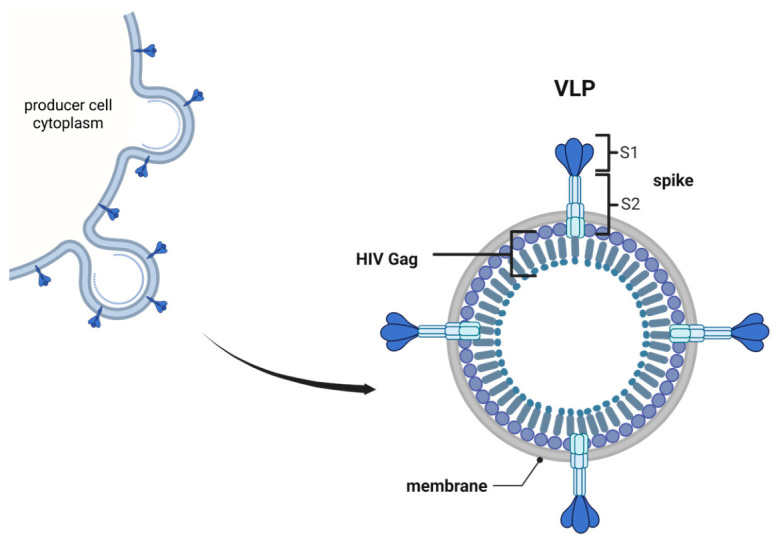
Schematic illustration of the assembly of Gag proteins at the cell membrane and the budding process of HIV VLPs decorated with SARS-CoV-2-derived spike proteins (**left**). The composition of VLPs with incorporated spike proteins and their subunits S1 and S2 are visualized (**right**). Created in BioRender. Pißarreck, M. (2026) https://BioRender.com/xjhuu6f (accessed on 19 May 2026).

VLPs derived from lentiviruses such as HIV-1 are characterized in depth and solely require the group-specific antigen (Gag) viral core protein for particle assembly and budding [14]. About 3500 Gag proteins [15,16] assemble at the host cell membrane at rigid microdomains called lipid rafts [17,18]. Budding of the particles from the VLP producer cell is supported by cellular endosomal sorting complexes required for transport (ESCRT) proteins [15,19]. The self-assembly of the precursor Gag proteins allows for continuous particle production from stable producer cells. The secretion of VLPs into the extracellular space enables the harvest from the cultivation media. Figure 1 shows the assembly and budding process and illustrates the composition of HIV VLPs displaying SARS-CoV-2 spike proteins consisting of subunits S1 and S2 separated upon cleavage by the cellular priming protease TMPRSS2 [20,21].

When produced in mammalian cells, VLPs are not the only membrane-enveloped particulates secreted into the cell culture supernatant. Non-viral extracellular vesicles (EVs) are cell-membrane-derived vesicles within a range of sizes from 45 to >1000 nm [22,23,24], overlapping with VLPs originating from the retrovirus family with diameters between 120 and 160 nm. Due to these similarities, VLP and EV separation can be difficult using methodologies frequently used in the purification of proteinaceous biologics such as ion exchange, multi-angle light scattering (MALS) and size exclusion chromatography [25,26,27]. Although also pursued as independent vaccine platforms, EVs are considered contaminants in VLP production, contributing to the host cell protein (HCP) and DNA (HCD) burden in VLP preparations [28].

As all membrane-enveloped viral particles, lentiviral VLPs can be pseudotyped, i.e., decorated with heterologous membrane-anchored proteins such as envelope proteins of other donor viruses. Such VLPs displaying antigen proteins in their natural structure elicit a robust neutralizing antibody (nAb) response upon vaccination, protecting from productive infection of the envelope-providing parental virus. Higher and more repetitive decoration density of the displayed proteins was demonstrated to increase immunogenicity and vaccine efficacy [29,30,31]. Decoration density is strongly influenced by the characteristics of the envelope protein, particularly by the transmembrane region (TMR) and cytoplasmic tail (CT) [32,33,34]. Alteration of the TMR—due to its proximity—is associated with a high likelihood of impairing the structural integrity of the membrane-anchored antigen ecto-domain, potentially resulting in a loss of function likely associated with a diminished potency to expose neutralization-sensitive epitopes and its ability to elicit a nAb response. Consequently, this proof of concept (POC) study focused on CT engineering to enhance decoration density while preserving the antigen structure of spike protein (S) variants derived from SARS-CoV-2.

We generated a panel of expression constructs encoding for different spike protein variants aiming at improving the antigens’ utility as a component in VLP vaccine prototypes. The truncated wildtype (wt) protein S-Δ19 was previously described to be efficiently incorporated into lentiviral vector particles and to mediate efficient gene transfer into human cell lines, proving the spike protein’s full functionality including receptor recognition and fusogenicity [21,35,36]. Variant S-HIV-1 encompasses the 154-amino-acid-long CT of the transmembrane (TM) envelope protein of HIV-1. This variant was designed because the CT interacts with a portion of the Gag protein during particle assembly, enabling active incorporation into VLPs [37,38,39]. The truncated CT of human platelet-derived growth factor receptor beta (PDGFRtr) was chosen in variant S-PDGFRtr due to its ability to facilitate a range of different protein displays on VLPs and vector particles formed by retroviral Gag used in a variety of previous projects in our research group and supported by the report of Goyvaerts et al. in 2012, demonstrating nanobody decoration of lentiviral vector particles [40]. Moreover, the very short CT of only eight amino acids was assumed to unlikely impact ectodomain folding. Spike variant S-GaLVΔR harbors the CT of the envelope of the retrovirus family member gibbon ape leukemia virus (GaLV) deleted for the so-called R peptide. The removal of this peptide by the viral protease renders the TM proteins fusogenically active [41]. This truncated CT of 16 amino acids was already demonstrated to mediate efficient incorporation into lentiviral particles for two other yet more closely related viral surface antigens, namely the envelope proteins of spleen necrosis virus (SNV) and murine leukemia virus (MLV) [42,43]. The panel of plasmid vectors was transfected in human embryonic kidney 293 cell derivates. Recombinant spike variants were analyzed for cellular and surface expression, receptor recognition and fusogenic activity using syncytia-formation assays and lentiviral vector pseudotype gene transduction into susceptible target cells overexpressing the SARS-CoV-2 receptor angiotensin converting enzyme 2 (ACE2) and the transmembrane serine protease 2 (TMPRSS2), which is pivotal for the priming of the spike protein [20,21]. Moreover, incorporation of spike proteins into VLPs and EVs as well as the exposure of a neutralizing epitope were assessed by conducting a Western blot analysis and an immuno-precipitation or VLP capture assay.

## 2. Materials and Methods

### 2.1. Plasmids

**Figure 2:**
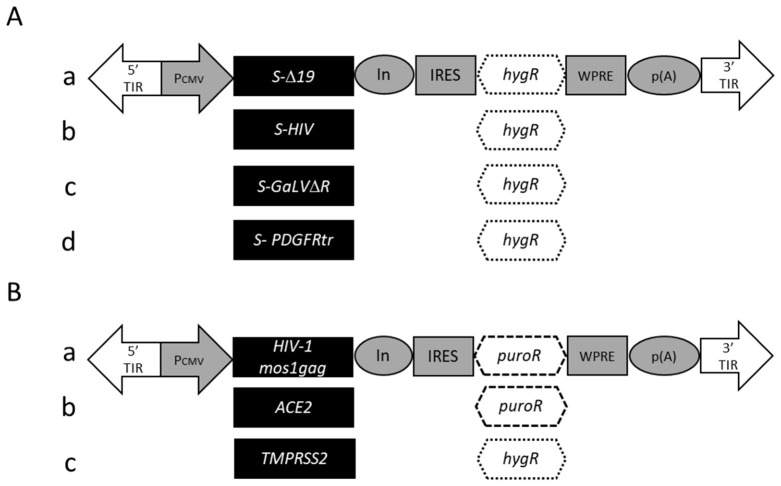
Schematic illustration of the utilized transposon expression vectors. The full cassettes shown in (**A**,**B**) share the same structural organization: a CMV promoter/enhancer element (P_CMV_) drives transgene expression followed by a synthetic intron (In). An internal ribosome entry site (IRES) couples the transgenes (black) to an antibiotic resistance gene—either hygromycin B (hygR) or puromycin (puroR)—on one full-length transcript. The woodchuck hepatitis posttranscriptional regulatory element (WPRE)—enhancing recombinant gene expression levels by improving the half-life of the full-length mRNAs—and a polyadenylation signal (p(A)) complete the 3′ end. Expression cassettes are flanked by terminal inverted repeats (TIRs) of Sleeping Beauty. (**A**) The constructs containing the four variants of the SARS-CoV-2 spike protein genes with modifications in the region encoding for the cytoplasmic tails (CTs) are illustrated: (a) encoding the wildtype SARS-CoV-2 spike protein with the CT truncated at the amino acid position 19 C-terminal of the transmembrane region, (b) substitution with the CT of HIV-1, (c) replacement with the CT derived from the gibbon ape leukemia virus (GaLV) transmembrane envelope protein deleted for the R peptide (GaLVΔR) and (d) CT derived from human platelet-derived growth factor receptor truncated to only 8 amino acids (PDGFRtr). (**B**) Vectors harboring the genes for (a) the mosaic mos1gag gene encoding for the precursor core proteins p55-Gag of HIV-1, (b) for the cellular protease TMPRSS2 and (c) for the SARS-CoV-2 receptor ACE2.

All spike variant expression constructs used in this study are *Sleeping Beauty* transposon vectors [44] to ease the future production of larger scales. The expression cassettes flanked by *Sleeping Beauty* terminal inverted repeats (TIRs) are depicted in Figure 2. The spike variant genes (Figure 2A) were coupled to a hygromycin B resistance gene to allow for the selection of stable pools of cells showing high expression levels. Briefly, variant SARS-CoV-2 wt spike Δ19 entails a codon-optimized Wuhan isolate 1 SARS-CoV-2 spike gene truncated to encode an only 18-amino-acid-long cytoplasmic tail (CT) [21,45]. Variants S-HIV, S-GaLVΔR, and S-PDGFRtr encode for the entire coronavirus spike protein ecto- and transmembrane region. Cytoplasmic domain coding sequences of an HIV-1 envelope gene (isolate NL4-3; GenBank accession no. AF324493.2), the truncated GaLV envelope encoding only 16 amino acid residues of the CT (GaLVΔR, GenBank accession no. AF055060.1; previously described by Tschorn et al. [43]), and the human platelet-derived growth factor receptor β (PDGFR; GenBank accession no. M21616.1) gene truncated to only eight amino acids were synthesized (GenScript, Rijswijk, The Netherlands; Eurofins, Hamburg, Germany) and used to substitute the truncated CT region of the coronavirus spike gene, resulting in variants S-HIV, S-GaLVΔR, and S-PDGFRtr.

For molecular cloning of the spike variant constructs, GeneArt™ Gibson Assembly HiFi Master Mix (Thermo Fisher Scientific, Waltham, MA, USA, #A46628) was used to fuse DNA fragments. The concept of Gibson Assembly is based on primers containing overhangs with homologous sequences to the target site; all primers contained such overhangs and are listed below. PCR was carried out employing Q5^®^ High-Fidelity DNA Polymerase (New England Biolabs, Ipswich, MA, USA, #M0491S). PCR amplicons were subjected to agarose gel electrophoresis and extracted from the gel with a Monarch^®^ Spin DNA Gel Extraction Kit (New England Biolabs, Ipswich, MA, USA, #T1120S) following the manufacturer’s instructions. A *Sleeping Beauty* expression vector encoding a hygromycin B resistance gene was opened in the multiple cloning site and linearized using the restriction enzyme FastDigest BstXI (Thermo Fisher Scientific, Waltham, MA, USA, #T1120S). The coding sequence of the CT-truncated (∆19) SARS-CoV-2 spike was amplified from the donor plasmid ([45]) using primers 1 and 2, as listed below in Table 1. The fragment was inserted into the *Sleeping Beauty* vector using Gibson Assembly. The S-HIV construct was generated from the same donor plasmid to amplify the coding sequence of the spike protein mentioned above but excluding the CT region (∆CT), employing primers 1 and 3. The HIV CT region was amplified from the respective donor plasmid template (GenScript synthesis, Rijswijk, The Netherlands) utilizing primers 4 and 5. The CT deleted spike ∆CT fragment and HIV CT fragment were fused by Gibson Assembly. The remaining two spike constructs S-GaLV∆R and S-PDGFRtr were constructed by instrumentalizing the same donor plasmid to amplify the coding sequence of spike ∆CT using forward primer 1. Due to the small size of the CTs, the coding sequences were included in the sequence of the reverse primers (GaLV∆R CT: primer 6; PDGFRtr CT: primer 7). The resulting amplicons contained the coding sequence of spike ∆CT fused to the respective CTs indicated and were inserted into the *Sleeping Beauty* recipient vector using Gibson Assembly.

The TMPRSS2 coding sequence was amplified from a plasmid gifted by Roger Reeves (Addgene, USA, plasmid #53887; http://n2t.net/addgene:53887, accessed on 19 May 2026; RRID:Addgene_53887) and inserted into the same *Sleeping Beauty*-derived expression construct carrying a hygromycin B resistance gene (Figure 2B, (c)). The ACE2 receptor gene (Sino Biological, Beijing, China; #10108-H05H) and the HIV-1 gag gene Mos1gag mediating the formation of VLPs upon expression as previously demonstrated using transmission electron microscopy [9] were cloned into a *Sleeping Beauty* expression construct carrying a puromycin resistance gene (Figure 2B, (a,b)).

### 2.2. Cells

Human embryonic kidney 293T (ATCC, Manassas, VA, USA, #CRL-1658) cells and their derivatives were grown in high-glucose Dulbecco’s Modified Eagle Medium (DMEM, Gibco, Thermo Fisher Scientific, Waltham, MA, USA, #11995065) supplemented with 10% Fetal Bovine Serum (Premium FBS, Gibco, Thermo Fisher Scientific, Waltham, MA, USA, #A5670701) and detached using TrypLE™ Express Enzyme (Gibco, Thermo Fisher Scientific, Waltham, MA, USA, #12604021). Suspension 293-F cells (Invitrogen, Waltham, MA, USA, #R79007) were grown in suspension in FreeStyle™ 293 Expression Medium (Gibco, Thermo Fisher Scientific, Waltham, MA, USA, #12338026) at 135 rpm. All cells were cultured at 37 °C with a humidified atmosphere at 5% for 293T and 8% CO_2_ 293-F cells, respectively. Cell counts were determined using fluidlab R-300 (Anvajo, Dresden, Germany).

Establishment of stable 293T cell lines expressing spike protein variants in concert with HIV Gag was achieved using 8 µg of the respective transposon constructs together with mRNA encoding for the SB100x Transposase using polyethylenimine (PEI; MW 40,000; Polysciences, Warrington, PA, USA), as previously described by van Heuvel et al. [46], followed by selection with increasing doses of hygromycin B and puromycin (Invivogen, Toulouse, France, #ant-hg-1 and#ant-pr-1, respectively), with final concentrations of 200 µg/mL and 10 µg/mL, respectively. Transient transfections of 293T cells were carried out using TransIT^®^-LT1 Transfection Reagent (Mirus Bio, Madison, WI, USA) following the manufacturer’s instructions.

The 293T cell pools stably overexpressing either ACE2 or ACE2 in concert with TMPRSS2 were generated following PEI-mediated transfection and as described by van Heuvel et al. [46], employing 16 µg of the above-mentioned cognate expression constructs. Subsequently, transfectants were selected using 10 µg/mL puromycin alone or together with 200 µg/mL hygromycin (both Invivogen, Toulouse, France, #ant-pr-1 and #ant-hg-1, respectively), resulting in the polyclonal cell lines 293T/ACE2 and 293T/ACE2/TMPRSS2, respectively.

### 2.3. Western Blotting and Preparation of VLPs and EVs

To detect recombinant protein expression, cells were pelleted at 100 g for 3 min. Cells were lysed using Pierce RIPA buffer (Thermo Fisher Scinetific, Waltham, MA, USA, #89900) for 30 min on ice. 4X Roti^®^LOAD 2 (Carl Roth, Karlsruhe, Germany, #K930.1) supplemented with DTT according to the manufacturer’s instructions was added to the lysates, followed by incubation at 95 °C for 15 min.

Upon passage through 0.45 µm filters, cell-free supernatants (CFSNs) were ultracentrifuged to pellet VLPs and EVs. For small volumes < 2 mL, particulates were pelleted at 21,630× *g* for 99 min at 4 °C using a MIKRO 200R benchtop centrifuge (Hettich, Tuttlingen, Germany). Larger volumes were subjected to 141,000× *g* for three hours using an Optima XE ultracentrifuge (Beckman Coulter, Brea, CA, USA). Small-scale samples were processed further as described above for the preparation of cell lysates. Pelleted particles resulting from large-scale volumes were resuspended in 15% (*w*/*v*) trehalose in PBS. Smaller volumes serving as samples for Western blot analysis were lysed as afore described.

Samples were loaded onto an MP TGX Stain-Free Gel (4–20% or 8–16%, Bio-Rad, Feldkirchen, Germany #4561094 and #4568104, respectively) and transferred to nitrocellulose membranes (Carl Roth, Karlsruhe, Germany, #200L.1). The membranes were blocked using 2% (*w*/*v*) milk powder in TBS-T (TBS-T/MP, 2 mM Tris-HCl, 15 mM NaCl, 0.05% Tween-20, pH 7.4) and stained using primary antibodies and a secondary HRP-conjugated antibody diluted in TBS-T/MP. SuperSignal™ West Pico PLUS Chemiluminescence-Substrate (Thermo Fisher Scientific, Waltham, MA, USA, #34580) was used to enable chemiluminescence. 1 µg/mL of rabbit anti-S2 primary antibodies (Invitrogen, Waltham, MA, USA, #PA1-41165), rabbit anti-HIV1 p55 + p24 + p17 primary antibodies (Abcam, Waltham, MA, USA, #63917) and chicken anti-rabbit IgG-HRP secondary antibodies (Invitrogen, Waltham, MA, USA, #A15987731-92-03113) were used in 1:1000 dilutions in this study. The software ImageJ 1.54 was employed to conduct densitometry to normalize the S2 protein to respective Gag amounts, enabling semi-quantitative analysis of spike protein incorporation efficiencies.

### 2.4. Detection of Cell-Surface Spike Protein Expression

For flow cytometric analysis of surface spike protein expression, cells were detached, washed in PBS supplemented with 2 mM EDTA and 0.5% (*w*/*v*) bovine serum albumin (BSA) and exposed to FITC-conjugated anti-S1 monoclonal antibodies (antibodies-online, #ABIN6953154) for detection of the S1 subunit. For the detection of the S2 unit, primary rabbit polyclonal anti-S2 antibodies (Invitrogen, Waltham, MA, USA, #PA1-41165) and Alexa Fluor 488-conjugated goat anti-Rabbit IgG secondary antibodies (Invitrogen, Waltham, MA, USA, #A-11008) were utilized using 1 µg of primary conjugated anti-S1 antibodies or unconjugated anti-S2 antibodies per 1.0 × 10^6^ cells at 4 °C for one hour. Samples incubated with rabbit anti-S2 antibodies were washed as described before and incubated with Alexa Fluor 488-conjugated anti-rabbit IgG secondary antibodies for another 30 min at 4 °C. All samples were analyzed employing a SH800s cell sorter (Sony Biotechnology, San Jose, CA, USA) and FlowJo (v10.9.0_CL) software.

### 2.5. Syncytia-Formation Assay

An amount of 3.0 × 10^5^ naïve 293T cells was seeded in 6-well dishes (Nunc, Munich, Germany) and co-transfected employing TransIT^®^-LT1 Transfection Reagent (Mirus, Madison, WI, USA) with the plasmid SB-GFP-IpW [47] expressing a reporter egfp gene under the control of a CMV promoter and either pS-Δ19-IhW, pS-HIV-IhW, pS-GaLVΔR-IhW, or pS-PDGFRtr-IhW. One day post-transfection, transfectants were detached using TrypLE™ Express Enzym (Gibco, Thermo Fisher Scientific, Waltham, MA, USA; #12604021), and 1.0 × 10^5^ cells transiently expressing spike protein variants and enhanced green fluorescent protein (EGFP) were mixed with 3.0 × 10^5^ naïve 293T, 293T/ACE2 and 293T/ACE2/TMPRSS2 target cells, respectively, and co-cultivated in 6-well dishes. Cells were co-cultured for 3 h and assessed for syncytia formation using an AXIO Vert.A1 fluorescence microscope (Zeiss, Oberkochen, Germany).

### 2.6. Generation of Lentiviral Vectors Pseudotyped with S Variant Proteins and Vector Titration

An amount of 3.0 × 10^6^ 293-F cells was seeded in 10 cm dishes in 5 mL media. Two hours post-seeding, cells were transfected with PEI with the packaging construct pCD/NL-BH*DDD (Addgene, Watertown, MA, USA, plasmid #17531; http://n2t.net/addgene:17531, accessed on 19 May 2026; RRID:Addgene_17531), the transfer vector pNL-EGFP/CMV/WPREdU3 (Addgene, Watertown, MA, USA, plasmid #17579; http://n2t.net/addgene:17579, accessed on 19 May 2026; RRID:Addgene_17579; gifted by Jakob Reiser) and the respective spike expression constructs at a ratio of 1:1:1. Following a five-hour incubation time, all media were exchanged for 10 mL fresh media. Three days post-transfection, 2.0 × 10^5^ target cells were seeded in 12-well dishes two hours prior to vector harvest. Vector-containing CFSNs were harvested and passaged through 0.45 µm syringe filters (ROTILABO^®^ PVDF, Carl Roth, Karlsruhe, Germany, #P667.1). Media was removed from target cells and replaced with 1 mL of vector-containing CFSN at different dilutions; 48 h later, transduced cells were detached and analyzed for EGFP expression using a SH800s cell sorter (Sony Biotechnology, San Jose, CA, USA) and FlowJo (v10.9.0_CL) software. Titers were calculated as described previously [48].

### 2.7. Immuno-Precipitation or VLP Capture Assay

Three million 293T cells were seeded per T175 flask, incubated for two hours and co-transfected using PEI and 10 µg Gag encoding plasmid, 8 µg of either spike variant, and 0.5 µg of the EGFP construct as a transfection control. Un-transfected cells (Mock) and cells only co-transfected with the Gag and EGFP plasmid served as negative controls. Two days post-transfection, CFSNs were harvested and ultracentrifuged as described above. Particle pellets were resuspended in 100 µL trehalose buffer (15% (*w*/*v*) trehalose in PBS). Aliquots of 4 µL of all samples were subjected to a Western blot analysis employing Gag-specific antibodies to estimate particle amounts. The Dynabeads™ Protein G Immunoprecipitation Kit (Invitrogen, Thermo Scientific, Waltham, MA, USA, #10007D) was used according to the manufacturer’s instructions and previously described in detail [49]. Briefly and per sample, magnetic beads were coated with 3.3 µg nAbs directed against the SARS-CoV-2 spike protein RBD of the strain Wuhan-Hu-1 (recombinant rabbit monoclonal antibody HL1003; Invitrogen, Thermo Scientific, Waltham, MA, USA, #MA5-46917). Unbound antibodies were removed by repeated washing, and 20 µL of particle-containing samples was added and incubated overnight. Unbound particles were removed by washing, and bound VLPs were eluted in 25 µL elution buffer. The eluates were supplemented with 15 µL PBS and 15 µL Rotiload/DTT and incubated at 95 °C for 15 min. A volume of 4 µL was used for Western blot analysis using Gag-specific antibodies as described above.

## 3. Results

### 3.1. Cellular Expression and Cell-Surface Display of SARS-CoV-2 Spike Variants

Spike protein variant genes were molecularly cloned into the *Sleeping Beauty* transposon vector SB-IhW, as illustrated in Figure 2. All encoded protein variants share the entire ecto- and transmembrane spanning domain of the SARS-CoV-2 spike protein, serving as a model for viral antigen expression. The variant S-Δ19 entails a truncated CT of the SARS-CoV-2 spike upon introduction of a stop codon at the 19th amino acid (aa) codon of the CT. Two other spike variants also encompass short CTs stemming from the mature gibbon ape leukemia virus (GaLV) missing the R peptide (GaLVΔR; 16 aa) and truncated human platelet-derived growth factor receptor (PDGFRtr; 8 aa). In contrast, variant S-HIV harbors a comparably long CT of 154 aa originating from HIV type 1.

**Figure 3:**
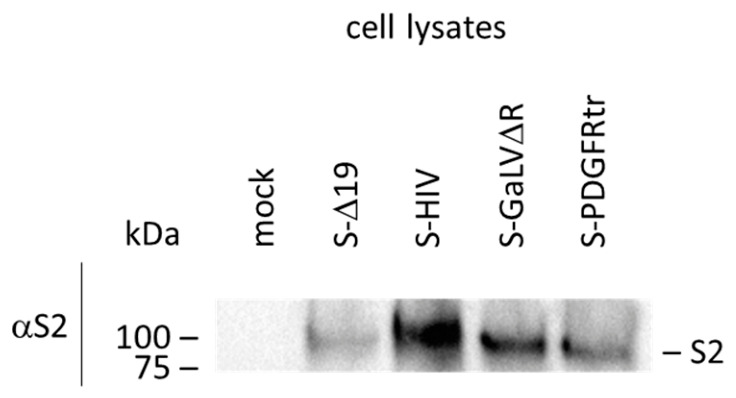
Western blot analysis of lysates from 293T cells transiently transfected with constructs encoding for the four spike variants as indicated using S2-specific primary antibodies. Naïve 293T cells served as negative control (mock). The positions of a molecular weight marker are indicated in kilodalton (kDa).

To assess the expression of all four spike variants, 293T cells were seeded and transfected with plasmids encoding either spike variant together with an EGFP encoding reporter construct serving as a transfection control. Two days post-transfection, confluent cell populations showing comparable transfection efficiencies of about 80% were harvested, lysed and subjected to Western blot analysis using polyclonal S2-specific primary and HRP-conjugated secondary antibodies. Antibodies directed against the S2 subunit were chosen since the S1 subunit may shed during sample preparation and prior to lysis of cells, while the S2 subunit is stably membrane-anchored. As indicated in Figure 3, expression of all four spike variants was readily detected. No S2 proteins were detected in lysates of naive 293T cells serving as negative controls. As expected, the S-HIV proteins revealed a higher molecular weight, as its CT is significantly longer than the CT of the other remaining variants. In summary, all four constructs were shown to mediate cellular expression of the cognate spike proteins.

**Figure 4:**
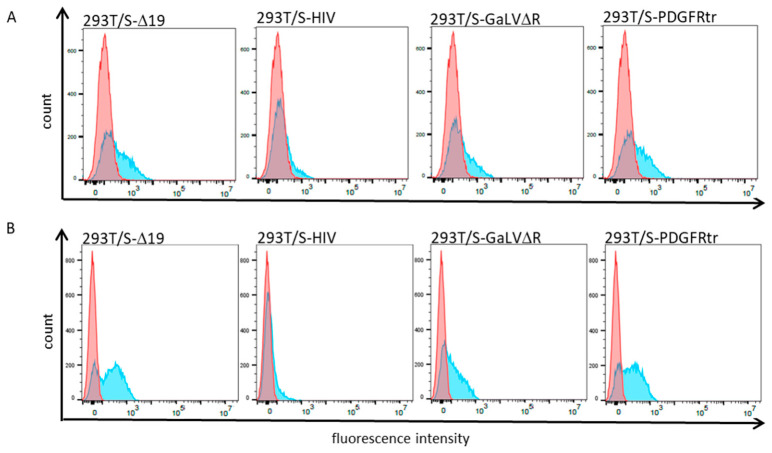
Flow cytometric analysis of the surface expression of the spike variants on transiently transfected 293T cells. Transfected cells are shown in blue, a naïve control sample in red. All samples were incubated with either (**A**) FITC-conjugated anti-S1 antibodies used to detect the S1 subunit of the spike protein or (**B**) rabbit anti-S2 primary antibodies and Alexa Fluor 488-conjugated goat anti-Rabbit IgG secondary antibodies to detect the S2 unit of the spike protein.

To examine whether the spike protein variants were also expressed on the cell surface, 293T cells were again transiently and individually transfected with the respective constructs in the absence of any other plasmid. Two days post-transfection, cells were detached, resuspended and exposed to spike-specific antibodies. Naïve 293T cells served as negative controls. As visible in Figure 4, histograms of all transfected cell populations—and in contrast to naïve cells—were demonstrated to cell-surface express reactive SARS-CoV-2 spike antigens. Furthermore, two different antibodies were used to detect either the S1 subunit prone to shedding but entailing the RBD or the stably membrane-anchored S2 subunit of the spike protein. As expected, S2 subunit-positive cells (Figure 4B) were detected at higher quantities. While the three protein variants S-Δ19, S-GaLVΔR and S-PDGFRtr showed comparably high surface expression, densities of S-HIV were considerably lower regardless of which subunit was targeted.

### 3.2. All Spike Variants Mediate Syncytia Formation

To investigate whether the engineered spike proteins retained their functional phenotype and thus were still able to bind the SARS-CoV-2 receptor angiotensin converting enzyme 2 (ACE2) as well as reveal fusogenicity, a panel of syncytia-formation assays were performed by co-culturing non-fluorescent target cells with cells co-expressing green fluorescence protein and the respective spike variant. If cell-to-cell fusion occurred, larger green fluorescent cells with multiple nuclei should be observable using fluorescence microscopy, demonstrating spike-mediated receptor binding and fusogenicity. 293T cells were again co-transfected with the respective spike variant expression constructs and an EGFP reporter construct. The following day, transfectants were detached and suspended with either naïve 293T cells or derivate cells stably expressing ACE2 alone (293T/ACE2) and in concert with the protease TMPRSS2 (293T/ACE2/TMPRSS2), respectively. TMPRSS2 was shown to prime the spike protein by cleavage at the S1/S2 site, and thus improves spike-mediated cell entry through fusion following receptor binding [20,21].

**Figure 5:**
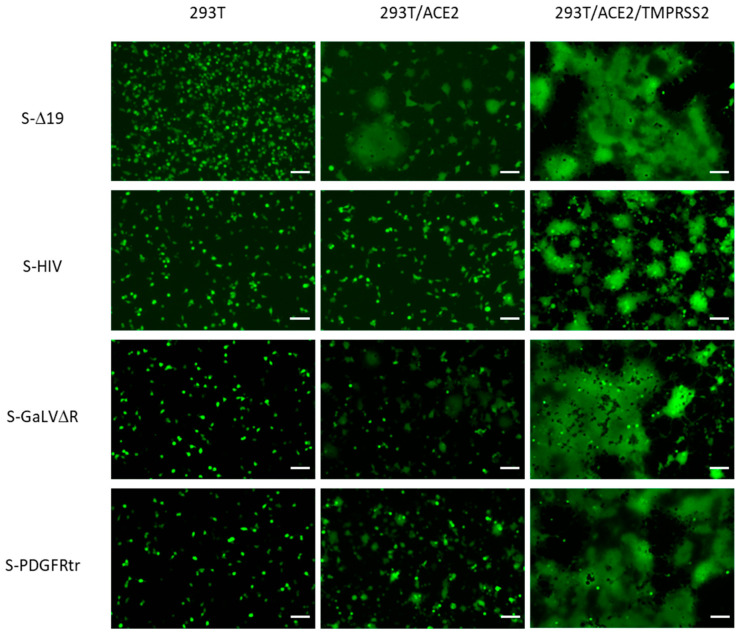
Syncytia-formation assay to examine the fusogenicity of spike protein variants. The 293T cells transiently co-transfected to express the respective spike protein variants indicated and EGFP were mixed and co-cultured with naïve 293T, 293T/ACE2 and 293T/ACE2/TMPRSS2 cells, respectively. Fluorescence microscopic images were taken after 3 h of co-cultivation. The scale bar indicates 100 µm.

The individual co-cultures were seeded, and images of fluorescent microscopic analysis were taken three hours later. As visualized in Figure 5, no syncytia were detected upon co-culture with naïve 293T cells. In contrast, some syncytia were observed in co-cultures with 293T/ACE2 cells. While the S-Δ19 protein mediated the formation of large syncytia at a high frequency, only a small and a much lower number of syncytia was observed upon expression of the other three spike variants. Strikingly, expression of all four protein variants resulted in profound numbers of large syncytia when co-cultured with 293T/ACE2/TMPRSS2 cells. However, S-HIV-mediated syncytia were visually smaller compared to the other three spike proteins.

### 3.3. All Spike Variants Form Transduction-Competent Pseudotype Lentiviral Vector Particles

In order to obtain more quantifiable data and to assess whether the spike variants also enable receptor recruitment and membrane fusion when displayed on virus-derived particles, lentiviral pseudotype vector particles were generated. Therefore, 293-F cells were transiently co-transfected with the respective spike expression constructs and second-generation HIV-based packaging and transfer vector constructs. The latter encompass an egfp reporter gene, allowing for the easy detection of transduced target cells using flow cytometric analysis. Three days post co-transfection, CFSNs were titrated in susceptible 293T/ACE2 and 293T/ACE2/TMPRSS2 target cells.

As shown in Table 2, all spike variants facilitated the formation of transduction-competent lentiviral pseudotype vectors and mediated gene transfer into susceptible target cells. In general, vector titers in 293T/ACE2/TMPRSS2 cells were considerably higher as compared to 293T/ACE2 target cells. Using naïve 293T target cells yielded such low titers not even quantifiable for HIV-1 (S-Δ19) vectors, mediating gene transfer efficiencies below the threshold of detection of <1.0 × 10^3^ transducing units per mL (TU/mL). The truncated S-Δ19 variant achieved the by far highest titers of up to >1.0 × 10^6^ TU/mL. In contrast and employing the variant S-HIV, the lowest titers were obtained, reaching a maximum of 1.90 × 10^4^ TU/mL in 293T/ACE2/TMPRSS2 cells. Variants S-GaLVΔR and S-PDGFRtr revealed overlapping yield ranges of 7.69 × 10^3^ to 2.45 × 10^4^ and 1.23 × 10^4^ to 5.71 × 10^4^ TU/mL.

### 3.4. Incorporation of Spike Protein Variants into VLPs and EVs

**Figure 6:**
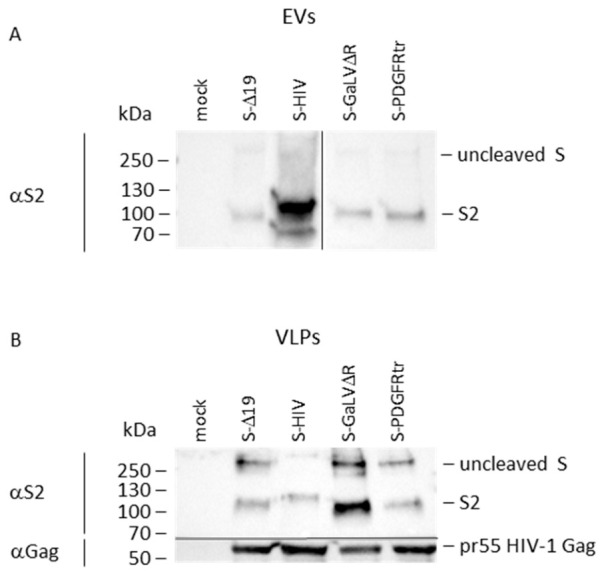
Detection of incorporated spike proteins in (**A**) extracellular vesicles (EVs) and (**B**) HIV Gag VLPs using Western blot analysis. The molecular weight marker positions are indicated on the left. Samples originating from naïve 293T cells served as negative controls (mock). (**A**) Pellets of EVs produced by 293T cells transiently expressing only the spike variants, detected with S2-specific antibodies. The images shown result from one membrane. A middle lane containing a molecular weight standard was cut from the image as indicated. (**B**) Spike protein variants decorated on VLPs (upper panel; uncleaved and processed S2) detected with S2-specific antibodies and Gag proteins (lower panel) using antibodies directed against the HIV core precursor protein. The membrane was cut just below the 70 kDa molecular weight marker protein after blotting and processed separately. Densitometric analysis of the respective S2 spike protein variant signal intensities normalized to the cognate Gag mounts resulted in relative values of 0.38 for SΔ19, 0.21 for S-HIV, 1.73 for S-GaLVΔR and 0.30 for S-PDGFRtr.

Intending to examine the incorporation efficiencies of spike proteins in EVs, 293T cells were transiently transfected with the respective expression constructs. CFSNs were harvested and subjected to ultracentrifugation, and resultant pellets were characterized using S2-specific primary and HRP-coupled secondary antibodies in a Western blot analysis. As illustrated in Figure 6A, all spike variants were readily detected but with much lower quantities for the uncleaved spike as compared to the S2 proteins. Strikingly, EVs were demonstrated to harbor large amounts of S-HIV proteins, while in contrast, only comparably small but similar amounts of all other three protein variants were detectable.

To access incorporation efficiencies of the spike variants into VLPs, 293T cells were co-transfected with the transposon vectors encoding for the HIV mos1gag Gag gene and the respective spike protein variant in concert with mRNA encoding the highly active transposase SB100x. Stable cell lines were established upon selection using antibiotics. CFSNs were harvested from the supernatant of stable producer cell lines, and naïve 293T cells served as negative controls (mock). Samples were ultracentrifuged and the pellets lysed and subjected to Western blot analysis using S2- and HIV-1 Gag-specific primary antibodies, respectively, followed by HRP-conjugated secondary antibodies.

As visualized in Figure 6B, comparable amounts of pr55 Gag precursor proteins were detected using specific antibodies demonstrating similar amounts of VLPs in all samples originating from stable producer cells. In contrast, diverse amounts of spike protein variants were detected, both the uncleaved spike and S2 proteins. The S-GaLVΔR proteins appeared to show superior incorporation efficiency as compared to all other variants. Variant S-HIV revealed the smallest amounts of proteins incorporated into VLPs. The S2 spike protein variant signal intensities were normalized to the related amounts of Gag and resulted, upon semi-quantitative densitometric analysis, in relative values of 0.38 for SΔ19, 0.21 for S-HIV, 1.73 for S-GaLVΔR and 0.30 for S-PDGFRtr.

### 3.5. All Spike Variants Expose Neutralization-Sensitive Epitopes on VLPs

**Figure 7:**
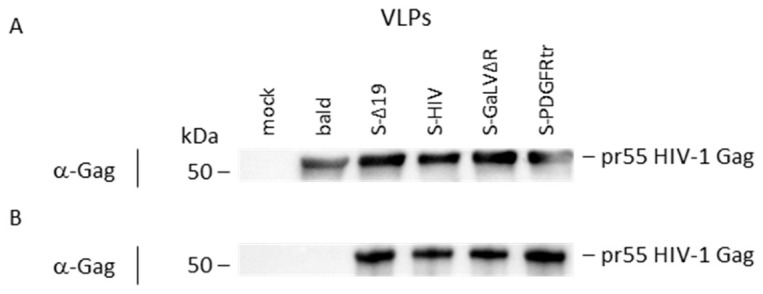
Detection of HIV core proteins employing Gag-specific antibodies in (**A**) particle pellet suspension volumes as used for capture assays. (**B**) Eluates of immuno-precipitated VLPs decorated with indicated spike protein variants from beads coated with nAbs directed against an epitope in the RBD. Samples from naïve 293T cells (mock) and 293T cells transiently expressing only Gag, and thus producing undecorated (bald) particles, served as negative controls.

In the last experiment of this study, we examined whether the spike protein variants were still exposing neutralization-sensitive epitopes and thus were able to elicit a corresponding neutralizing B cell response in vaccinees. Two days post-transient-transfection, comparable transfection efficiencies were observed by fluorescent microscopic detection of EGFP-expressing cells. Particle-containing CFSNs were harvested from 293T cells expressing an EGFP reporter construct, HIV Gag and respective spike variants. Samples of mock-transfected and only Gag and EGFP-expressing cells, the latter producing bald VLPs, served as negative controls. Samples were ultracentrifuged, and resultant particle pellets were resuspended. Aliquots of these samples were examined in a Western blot analysis using Gag core protein-specific antibodies to confirm comparable amounts of VLPs, as visible in Figure 7A.

Protein G-coated magnetic beads were incubated with monoclonal nAbs targeting the RBD within the spike subunit S1. Subsequently, the nAb-covered beads were mixed with the respective particle suspensions. After repeated washing, eluates were examined employing Gag-specific antibodies in a Western blot analysis. As shown in Figure 7B, no Gag proteins were detectable in negative controls of mock-transfected cells or bald, i.e., spike protein negative, particles, demonstrating the negligible background binding activity of VLPs and nAb-coated beads. In contrast, all other samples containing spike protein variant decorated VLPs revealed comparable amounts of the viral core protein Gag, proving the display of the neutralizing-sensitive epitope within the RBD targeted by the nAb employed in the immuno-precipitation or VLP capture assay.

## 4. Discussion

VLPs are potent vaccine components due to their high morphological similarity to infectious virus particles eliciting a strong cellular and humoral immune response. Membrane-enveloped VLPs such as HIV-1-derived particulates allow not only for the display of their cognate surface proteins but also of heterologous antigens of other donor viruses. The density of displayed antigens is paramount to the potency of a VLP vaccine candidate. In this proof of concept (POC) study, HIV VLPs decorated with the spike protein of SARS-CoV-2 were chosen as a prototype or model aiming at improving surface antigen density by engineering of the CT.

Expression vectors were generated encoding for the truncated spike protein S-Δ19 and CT-substituted variants S-GaLVΔR, S-HIV and S-PDGFRtr and were demonstrated to be readily expressed in transfected 293T cells. Using S1- and S2-specific antibodies, flow cytometric analysis revealed comparable cell-surface expression of variants S-Δ19, S-GaLVΔR, and S-PDGFRtr with the exception of S-HIV proteins achieving notably lower levels. The very long CT of the HIV envelope protein has been reported to limit surface expression of these proteins [50] by clathrin-mediating endocytosis. The membrane-proximal YXXF motif and a dileucine at the very C-terminus of the CT interact with the clathrin-associated adaptor protein complex (AP-2) [51,52,53,54,55]. However, this is to our knowledge the first time this restricted surface density was shown to be transferable to heterologous, namely, SARS-CoV-2 spike proteins.

All variants were demonstrated to induce syncytia formation when expressed in 293T cells and upon co-cultivation with cells recombinantly overexpressing ACE2 alone and in concert with TMPRSS2, while failing to fuse with naïve 293T cells. This indicated the low-level expression of at least the SARS-CoV-2 receptor ACE2 in 293T cells, later also substantiated by the inferior susceptibility to lentiviral pseudotype vector-mediated gene transfer. The truncated variant S-Δ19 revealed the highest fusogenicity, already leading to the formation of large syncytia upon co-cultivation with 293T/ACE2 cells, followed by S-GaLVΔR and S-PDGFRtr. These variants with considerably short CTs of 16 and 8 amino acid residues thus only lowered the fusogenicity as compared to the S-Δ19, suggesting a limited influence on the conformation of the transmembrane and ecto-domain. In contrast, S-HIV proteins showed the weakest induction of syncytia, also observed by the smallest size of the fused poly-nuclear cells after co-incubation with 293T/ACE2/TMPRSS2 cells. Whether this is caused by lower fusogenicity, and thus a stronger impairment of the structural conformation of the spike variant with a CT of 154 amino acids, or alternatively, is simply a result of the lower cell-surface expression remains elusive. In conclusion, the syncytia-formation assays confirmed for all variants examined that receptor recruitment and fusogenicity were still preserved. This makes it feasible to expect the elicitation of neutralizing antibodies (nAbs) upon potential future vaccination studies; as such, nAbs directed against SARS-CoV-2 spike proteins most frequently target the receptor binding domain (RBD) in S1 [56,57].

To examine the spike variants’ ability to recruit the host cell receptor ACE2 and to mediate membrane fusion when incorporated in virus-derived particles, lentiviral vectors were pseudotyped with the spike variant proteins. Considering a standard deviation for only two titers per pseudotype vector of poor value, only the titration in 293T/ACE2/TMPRSS2 target cells delivered robust data. Variant S-Δ19 enables vector yields more than twenty-fold higher as compared to S-PDGFRtr, closely followed by S-GaLVΔR, with S-HIV being least efficient. The observed titers therefore confirmed the findings from the syncytia-formation assay. However, these findings do not directly mirror the decoration density of spike proteins on lentiviral vector particles yet prove the incorporation of all spike variants into HIV-derived particles. Consequently, the incorporation efficiency of spike proteins was assessed for both VLPs and EVs.

While variants S-Δ19, S-PDGFRtr and S-GaLVΔR were barely detectable as uncleaved spike proteins in EVs and only weak signal intensities were observed for processed S2-variants, the S2 domain of S-HIV was visualized in large quantities with a molecular weight of >100 kDa. The smaller proteins of about 70 kDa may represent incompletely glycosylated protein or degradation products. HIV envelope proteins were described to be incorporated into EVs, while the domains facilitating this have not yet been mapped [58]. However, our data presented here indicate that the CT of HIV transferred the enhanced incorporation into EVs to the TMR and ecto-domain of SARS-CoV-2 spike proteins.

In contrast, comparable amounts of VLPs showed large amounts of spike variant S-GaLVΔR, suggesting the most efficient incorporation, both as uncleaved S and processed S2. S-Δ19 and S-PDGFRtr were yet still slightly elevated as compared to S-HIV. Although the CT of HIV most likely interacted with the matrix subunit of Gag mediating active incorporation into Gag-formed VLPs, the enhanced endocytosis of S-HIV proteins as aforementioned resulted in the low amounts of chimeric spike proteins [50]. Moreover, it needs to be explicitly mentioned that the concentration of particles using ultracentrifugation does not facilitate the distinction of VLPs and EVs. It therefore cannot be excluded that the spike proteins co-detected with Gag did not exclusively result from decorated VLPs but originated from considerable amounts of contaminating EVs displaying spike proteins. This holds particularly true for the S-HIV proteins found in large quantities in EVs, while the only low amounts of the other variants appear to be negligible.

In contrast to S-HIV proteins, the passive—i.e., Gag-independent—incorporation into VLPs was more efficient. Semi-quantitative densitometric Western blot analysis revealed the superior incorporation of S-GaLVΔR proteins into VLPs, exceeding the decoration efficiency of S-Δ19 by more than four-fold and S-PDGFRtr by more than five-fold. As the CT of variant S-GaLVΔR was previously shown to mediate the superior incorporation of heterologous viral surface antigens into lentiviral vector particles [42,43], it could be assumed that this was owing to the high-density localization in membrane regions where the budding of particles occurs, namely, lipid rafts [17,18].

To qualify as future vaccine components, spike proteins displaying VLPs need to be capable of eliciting neutralizing humoral responses in vaccinees. Prior to a potential pre-clinical testing in a small animal model and as a last in vitro evaluation, the binding of a nAb to the RBD in the spike proteins was assessed in an immuno-precipitation experiment or VLP capture assay. Strikingly, all examined spike protein variants exposed the neutralization-sensitive epitope. It is thus feasible to assume that further epitopes are probably also conserved, rendering all variants of potential value as vaccine prototype components. However, in vivo testing will be necessary to validate this.

In conclusion, our data strongly suggest that the replacement of CT domains of SARS-CoV-2 spike proteins with homologous regions of selected viral and non-viral membrane proteins does not completely abrogate the protein’s functionality. Protein variants were still (i) cell-surface expressed, while (ii) receptor recognition and fusogenicity remained preserved, (iii) a neutralizing-sensitive epitope was still available to elicit respective antibody responses upon immunization, and (iv) the chimeric spike proteins were all incorporated into VLPs and (v) were detectable in in EVs. The CT of HIV mediated the highest incorporation efficiency into EVs and the lowest in VLPs. The truncated and R peptide-deleted CT of GaLV appeared to mediate the highest decoration density on VLPs while being just detectable in EVs, rendering the S-GaLV∆R protein the most promising candidate as a potential VLP vaccine component. We thus anticipate that the results of our POC will be of utility in future chimeric VLP prototype vaccine candidate developments. This could include other viral or non-viral antigens such as neoantigens presented on tumor cells.

## Figures and Tables

**Table 1:** Primer sequences used in the construction of the spike variant expression constructs.

**1**	FOR_spike ∆19 5′-TCGTACGTTAACATAGATAACTGATCCAGTGTGACCATGTTCGTGTTTCTGGTGCTGCT
**2**	REV_spike ∆19 5′-AACAGCTGGCCCTCGCAGACAGCGAATTAATTCCAGCACACTGGCTAGCAGCAGCTG CCACAGCT
**3**	REV_spike ∆CT for HIV-1 CT assembly 5′-CCTAACTCTATTCACGCAACACAGCATGATTGTGACC
**4**	FOR_HIV CT 5′-ATCATGCTGTGTTGCGTGAATAGAGTTAGGCAGGGATATTCACCA
**5**	REV_HIV CT 5′-CCTCGCAGACAGCGAATTAATTCCAGCACACTATTATAGCAAAATCCTTTCCAAGC
**6**	REV_spike ∆CT (including GaLV∆R CT in bold letters) 5′-CCTCGCAGACAGCGAATTAATTCCAGCACACT**ATTACAGAATTTTAACTGCACTTATC** **CTATCATTGATGAATTGAACTAACTT**GCAACACAGCATGATTGTGACCATCA
**7**	REV_spike ∆CT (including PDGFRtr CT in bold letters) 5′-CCTCGCAGACAGCGAATTAATTCCAGCACACTA**TTAACGTGGCTTCTTCTGCCAAAG** **CAT**GCAACACAGCATGATTGTGACCATCACGATGGCAATCAGTCCGGCGATAA

**Table 2:** Lentiviral HIV-1-derived vectors pseudotyped with respective spike variants and obtained titers upon titration in 293T/ACE and 293T/ACE2/TMPRSS2 target cells resulting from three independent experiments (replicates I, II, III). Titers are shown as transducing units per mL (TU/mL). Mean values with standard deviations (SD) are indicated; not detectable (n.d.) refers to a detection limit of 1% of transduced EGFP-positive cells equal to 1.0 × 10^3^ TU/mL; not applicable (N/A).

Target Cell Line	Spike Variant	Titer (TU/mL)				
		I	II	III	Mean	SD
293T/ACE2	S-Δ19	1.64 × 10^5^	4.58 × 10^4^	1.96 × 10^4^	**7.65 × 10^4^**	5.92 × 10^4^
	S-HIV	8.34 × 10^3^	n.d.	n.d.	**N/A**	N/A
	S-GaLVΔR	7.25 × 10^3^	9.05 × 10^3^	n.d.	**8.15 × 10^3^**	N/A
	S-PDGFRtr	2.18 × 10^4^	4.03 × 10^3^	n.d.	**1.29 × 10^4^**	N/A
293T/ACE2 /TMPRSS2	S-Δ19	1.23 × 10^6^	6.12 × 10^5^	3.16 × 10^5^	**7.21 × 10^5^**	3.11 × 10^5^
	S-HIV	1.90 × 10^4^	5.54 × 10^3^	1.99 × 10^3^	**8.84 × 10^3^**	6.73 × 10^3^
	S-GaLVΔR	2.45 × 10^4^	1.04 × 10^4^	7.69 × 10^3^	**1.42 × 10^4^**	7.03 × 10^3^
	S-PDGFRtr	5.71 × 10^4^	3.10 × 10^4^	1.23 × 10^4^	**3.35 × 10^4^**	1.30 × 10^4^

## Data Availability

The original contributions presented in this study are included in the article. Further inquiries can be directed to the corresponding author.

## Abbreviations

The following abbreviations are used in this manuscript:

aa	amino acids
ACE2	angiotensin-converting enzyme 2
BSA	bovine serum albumin
CFSN	cell-free supernatant
CT	cytoplasmic tail
DMEM	Dulbecco’s Modified Eagle Medium
EGFP	enhanced green fluorescent protein
EV	extracellular vesicle
GaLV	gibbon ape leukemia virus
GaLVΔR	truncated GaLV envelope encoding only 16 amino acid residues of the CT
hygR	hygromycin B resistance gene
In	synthetic intron
IRES	internal ribosome entry site
kDa	kilodalton
MLV	murine leukemia virus
nAbs	neutralizing antibodies
P_CMV_	CMV promoter/enhancer element
PDGFRtr	truncated human platelet-derived growth factor receptor β
PEI	polyethylenimine
p(A)	polyadenylation signal
puroR	puromycin resistance gene
S(2)	(subunit 2 of) SARS-CoV-2 spike protein
SNV	spleen necrosis virus
TBS-T	Tris-buffered saline with Tween-20
TIRs	terminal inverted repeats
TMPRSS2	transmembrane serine protease 2
TU/mL	transducing units per mL
VLP	virus-like particle
WPRE	woodchuck hepatitis posttranscriptional regulatory element
wt	wildtype
